# Chemogenetic Schwann cell activation impairs early myelination and triggers adult demyelination in the peripheral nervous system

**DOI:** 10.3389/fncel.2026.1771951

**Published:** 2026-02-16

**Authors:** Jazmin G. Corral, Veronica T. Cheli, Diara A. Santiago-Gonzalez, Karishma G. Kedari, Pablo M. Paez

**Affiliations:** Department of Pharmacology and Toxicology, Institute for Myelin and Glia Exploration, Jacobs School of Medicine and Biomedical Sciences, The State University of New York, University at Buffalo, Buffalo, NY, United States

**Keywords:** calcium channels, calcium signaling, chemogenetics, DREADDs, hM3Dq, myelin, Schwann cells

## Abstract

Schwann cells (SCs), the myelin-forming glia of the peripheral nervous system (PNS), are essential for nerve development and maintenance; however, the contribution of Ca^2+^ signaling to their maturation and long-term stability remains poorly understood. Here, we present a chemogenetic approach to selectively manipulate Gq-mediated Ca^2+^ signaling in SCs across developmental stages. By combining *Cre*-dependent expression of the excitatory DREADD hM3Dq with activation by clozapine-N-oxide, we achieved precise, temporally controlled stimulation of the canonical Gq–PLC–IP3–Ca^2+^ cascade. *In vitro*, hM3Dq activation in immature SCs elevated basal Ca^2+^ levels, amplified spontaneous oscillations, and suppressed voltage- and ligand-gated Ca^2+^ influx, completely blocking SC maturation and myelin protein expression without affecting survival or proliferation. *In vivo*, early postnatal activation severely impaired sciatic nerve myelination, resulting in thinner myelin sheaths, fewer myelinated axons, and abnormal Remak bundle organization. Conversely, activation in mature SCs induced progressive demyelination, axonal degeneration, and motor deficits in adult mice. Ultrastructural and biochemical analyses confirmed widespread myelin loss and reduced expression of key myelin proteins, accompanied by increased g-ratios and axonal pathology. These findings uncover a previously unrecognized, bidirectional role for sustained Gq signaling in SC biology—blocking developmental myelination and destabilizing mature myelin through Ca^2+^ dysregulation. Our study establishes excitatory DREADDs as a powerful tool for probing stage-specific signaling requirements in peripheral glia and highlights Ca^2+^ homeostasis as a critical determinant of PNS integrity, with implications for demyelinating neuropathies and regenerative therapies.

## Highlights

- Activation of hM3Dq disrupts Schwann cell Ca^2+^ signaling, delays sciatic nerve myelination, and disrupts Remak bundle structure during development.
- In adult mice, hM3Dq activation induces demyelination, axonal degeneration, and motor deficits.

## Introduction

Schwann cells (SCs) are the principal glial cells of the peripheral nervous system (PNS), where they play indispensable roles in nerve function and regeneration. By forming the myelin sheath around peripheral axons, SCs enable rapid electrical conduction and provide trophic support essential for neuronal survival and maintenance (Jessen and Mirsky, 2005, 2019). Beyond these functions, SCs act as first responders following nerve injury—clearing axonal debris and guiding the regeneration of damaged fibers (Mirsky et al., 2008; Vallejo et al., 2022). Through these diverse activities, SCs exert profound influence over sensory and motor pathways throughout life, making them critical for peripheral nerve health (Feltri et al., 2016).

Although recent studies have extensively explored the role of Ca^2+^ channels and receptors in oligodendrocyte development and CNS myelination (Paez and Lyons, 2020), the contribution of Ca^2+^ signaling to SC development and function in the PNS remains poorly understood. Emerging evidence suggests that Ca^2+^ signaling is a key mechanism by which SCs interact with neurons and regulate peripheral nerve physiology. SCs detect neuronal activity through purinergic (P2X/P2Y), muscarinic (M2, M3), and glutamate receptors, which trigger Ca^2+^ influx via voltage- and ligand-gated channels and release from intracellular stores through IP3-mediated pathways (Lyons et al., 1994; Fields and Burnstock, 2006; Baker, 2002). These Ca^2+^ transients influence essential processes such as SC proliferation, differentiation, myelination, and synaptic plasticity at neuromuscular junctions (Samara et al., 2013; Heredia et al., 2020). In myelinating SCs, Ca^2+^ signals propagate through paranodal regions and gap junctions, whereas in non-myelinating SCs they regulate K^+^ buffering and neurotransmitter secretion (Samara et al., 2013; Heredia et al., 2020). Dysregulation of these pathways—including altered purinergic signaling or store-operated Ca^2+^ entry—has been linked to peripheral neuropathies such as Charcot–Marie–Tooth disease (Vanoye et al., 2019). Collectively, these findings position Ca^2+^ signaling as a dynamic regulator of SC physiology, enabling adaptation to neuronal activity and maintenance of nerve integrity.

To investigate the functional role of Gq-mediated Ca^2+^ signaling in SCs, we used Designer Receptors Exclusively Activated by Designer Drugs (DREADDs)—chemogenetic tools that enable precise, cell-type–specific modulation of intracellular signaling via clozapine-N-oxide (CNO), an otherwise inert clozapine metabolite (Roth, 2016). Specifically, we expressed hM3Dq, an excitatory Gq-coupled receptor derived from the human muscarinic M3 receptor, in SCs. Developing SCs naturally express multiple Gq-coupled receptors—including purinergic (P2Y), muscarinic (M3), and lipid-sensing LPA and S1P receptors—that respond to axon-derived signals by generating Ca^2+^ transients, which regulate SC plasticity and developmental timing (Fields and Stevens, 2000; Anliker et al., 2013). Chemogenetic activation of Gq signaling via hM3Dq provides a powerful strategy to bypass receptor redundancy and compensatory mechanisms, enabling temporally controlled activation of the canonical Gq–PLC–IP3–Ca^2+^ cascade during defined developmental windows. This approach is ideally suited for dissecting stage-specific requirements for Ca^2+^ signaling during early myelination and in the adult PNS.

Our previous work in oligodendrocytes demonstrated that hM3Dq activation triggers Ca^2+^ release from intracellular stores and enhances Ca^2+^ influx, promoting oligodendrocyte progenitor proliferation while inhibiting maturation and myelin protein synthesis (Cheli et al., 2026). Here, we extend this approach to SCs and show that hM3Dq activation during early postnatal development significantly delays sciatic nerve myelination and reduces the density of mature SCs *in vivo*. Conversely, activation in mature myelinating SCs leads to myelin degradation, peripheral neurodegeneration, and impaired motor coordination in adult mice. These effects were accompanied by altered spontaneous Ca^2+^ oscillation amplitude and changes in specific Ca^2+^ channel activity. Together, these findings reveal a bidirectional role for hM3Dq-mediated Ca^2+^ signaling in SC development and function and establish excitatory DREADDs as a versatile tool for manipulating PNS myelination across developmental stages.

## Materials and methods

### Transgenic mice

All experimental animals were housed in the Roswell Park Division of Laboratory Animal Medicine vivarium. Procedures were approved by University at Buffalo’s Animal Care and Use Committee and conducted in accordance with the National Institutes of Health’s *Guide for the Care and Use of Laboratory Animals*. The following transgenic mice were obtained from The Jackson Laboratory: hM3Dq/mCitrine (JAX stock # 026220) and Sox10-i*Cre*ER^T2^ (JAX stock #027651). Experimental animals were generated by crossing the hemizygous hM3Dq/mCitrine line with hemizygous Sox10-i*Cre*ER^T2^ transgenic mice. For simplicity, conditional hM3Dq mice (hM3Dq^+/−^, Sox10-i*Cre*ER^T2+/−^) will be referred to as *Sox10-hM3Dq* for the remainder of this manuscript. In all the experiments presented in this work, mice of both sexes were used. To induce the expression of hM3Dq receptors in Sox10 expressing cells, *Cre* activity was induced by tamoxifen starting at postnatal day 2 (P2). Mice were intraperitoneal (IP) injected once a day for 5 consecutive days with 25 mg/kg tamoxifen (Sigma-Aldrich) dissolved in corn oil (Sigma-Aldrich). To activate hM3Dq receptors, P6 Sox10-hM3Dq mice were given either 1 mg/kg clozapine-N-oxide (CNO) (Hellobio), or vehicle (0.9% saline solution, BD) via IP injections once a day for 10 days. Sciatic nerves were collected at P10 and P15 for analysis. For experiments with adult mice, P50 Sox10-hM3Dq mice were injected intraperitoneally once a day for 5 consecutive days with tamoxifen (100 mg/kg). Then, at P60 these animals were given IP injections of 1 mg/kg CNO or vehicle (0.9% saline solution) once a day for 10 consecutive days and sciatic nerves were collected at P70, P80 and P90.

### Dorsal root ganglia (DRG) isolation and culture

The DRGs were harvested from Sox10-hM3Dq mouse embryos at embryonic day 13.5. For each embryo, 20 DRGs were dissected and placed into 1 mL of Leibovitz’s L-15 medium (Invitrogen). The tissues were centrifuged at 200 × g for 5 min and resuspended in 500 μL of 0.25% trypsin solution lacking EDTA (Invitrogen). Enzymatic digestion was carried out at 37 °C for 45 min. To halt trypsin activity, 500 μL of L-15 medium supplemented with 10% fetal bovine serum (FBS; Invitrogen) was added. The supernatant was discarded, and the cells were washed once more with 1 mL of L-15 medium containing 10% FBS, followed by another centrifugation at 200 × g for 5 min. The resulting cell pellet was gently triturated and resuspended in C-medium consisting of Minimum Essential Medium (MEM; Invitrogen) supplemented with 4 g/L D-glucose, 10% FBS, 2 mM L-glutamine, 50 ng/mL nerve growth factor (NGF), and gentamycin. A 150 μL aliquot of the cell suspension was seeded at the center of a 15 mm collagen and poly-L-lysine (PLL)-coated coverslip. Cultures were incubated overnight at 37 °C in 5% CO₂. The following day, the medium was replaced with Neurobasal medium (Invitrogen) containing B27 supplement (Invitrogen), 2 mM L-glutamine, 50 ng/mL NGF, and gentamycin. After 5 days in culture, the cells were transitioned back to C-medium, which was subsequently refreshed every other day. To induce *Cre*-mediated recombination, cultures were treated with 0.5 μM 4-hydroxytamoxifen (Sigma-Aldrich) for two consecutive days beginning on day 5 post-plating. To promote SC maturation and myelination, cultures were supplemented with 50 μg/mL ascorbic acid (Sigma-Aldrich) starting on day 7 post-plating. In parallel, cultures received either vehicle (0.9% saline solution) or 10 μM CNO for 14 days to activate the hM3Dq receptor.

### Calcium imaging

The DRG co-cultures were prepared from Sox10-hM3Dq mouse embryos at embryonic day 13.5, as described above. To induce hM3Dq expression in SCs, cultures were treated with 0.5 μM 4-hydroxytamoxifen for two consecutive days beginning on day 5 post-plating. On day 7, cells were transduced with an adenoviral vector encoding the Ca^2+^ indicator GCaMP6m (Ad-GCaMP6m; Vector Biolabs) at a multiplicity of infection (MOI) of 25 for 24 h. Following transduction, cultures were treated for 3 days with either vehicle (0.9% saline solution) or 10 μM CNO to activate hM3Dq signaling. Prior to imaging, cells were gently washed with sterile PBS and incubated in phenol red-free Hanks’ Balanced Salt Solution (HBSS; Gibco) containing Ca^2+^ and Mg^2+^, or in HBSS devoid of Ca^2+^ (Gibco). Ca^2+^ transients and resting Ca^2+^ levels were quantified at the single-cell level within SC somas, with data pooled from three independent biological replicates per condition. For each replicate, more than 100 cells were manually selected for analysis. GCaMP fluorescence was excited at 480 nm using a high-speed wavelength-switching system (Lambda DG4; Sutter Instruments). Emission was captured via a spinning disk confocal inverted microscope (Olympus IX83-DSU) equipped with a CCD camera (Hamamatsu ORCA-R2). Image acquisition and analysis were performed using MetaFluor software (Molecular Devices). To minimize photobleaching, both the excitation light intensity and sampling frequency were optimized for low exposure. Fluorescence measurements were acquired every 2 s for a total duration of 480 s (8 min). At 200 s into the recording, cells were stimulated with one of the following agents: ATP (100 μM), glutamate (100 μM), potassium chloride (K^+^ 50 mM), or acetylcholine (100 μM) (Sigma-Aldrich).

### Immunocytochemistry

Cells were rinsed with PBS and fixed with 4% paraformaldehyde (PFA) for 20 min at room temperature. Permeabilization was carried out using methanol for 10 min at room temperature. Immunostaining was performed following the procedure described by Cheli et al. (2023), with minor modifications. Briefly, fixed cells were incubated in blocking solution containing 0.1% Triton X-100, 1.5% bovine serum albumin (BSA), and 5% goat serum, followed by overnight incubation at 4 °C with the primary antibodies. Secondary antibody incubation was performed using fluorophore-conjugated antibodies (1:600; Jackson ImmunoResearch Laboratories). Nuclear staining was conducted using DAPI (Invitrogen), and samples were mounted with Aquamount (Lerner Laboratories). Fluorescent imaging was carried out using a spinning disc confocal microscope (Olympus IX83-DSU). Myelin internodes and marker-positive cells were quantified in at least three independent biological replicates per condition, with 10 randomly selected fields analyzed per replicate. Semi-automated cell quantification was performed using MetaMorph software (Molecular Devices). Quantification of myelin internodes was performed using ImageJ Fiji version 1.54p. Images were pre-processed using a Gaussian blur filter to reduce background noise, followed by thresholding to identify internodes. Objects smaller than 50 μm^2^ or with circularity more than 0.3 were excluded to eliminate nonspecific staining. Overlapping internodes that could not be accurately segmented were also excluded from analysis. DAPI staining was used to ensure the total number of cells was equivalent across experimental conditions. Primary antibodies: Ki67 (rat; 1:500; BD Biosciences), MAG (mouse; 1:1000; Abcam), MBP (mouse; 1:1000; Covance), P0 (chicken; 1:3000; Aves), Sox2 (mouse; 1:500; R&D Systems), Sox9 (rabbit; 1:500; Cell Signaling Technology), and Sox10 (rabbit; 1:1500; Cell Signaling Technology).

### Immunohistochemistry

Sciatic nerves were dissected from mice and placed in tubes containing 4% paraformaldehyde (PFA) in PBS for overnight fixation at 4 °C. Longitudinal cryosections (10 μm thickness) were prepared using a clinical cryostat (Leica Microsystems) and mounted onto Superfrost Plus microscope slides (Thermo Fisher Scientific). Tissue sections were incubated in blocking solution containing 2% Triton X-100, 1.5% bovine serum albumin (BSA), and 5% goat serum for 2 h at room temperature. Primary antibody incubation followed overnight at 4 °C. The next day, sections were rinsed with PBS and incubated with the corresponding secondary antibodies (1:400; Jackson ImmunoResearch Laboratories) for 2 h at room temperature. Nuclear staining was performed using DAPI (Invitrogen), and slides were mounted with Aquamount (Lerner Laboratories) following final PBS washes and gentle air drying. The primary antibodies used for immunohistochemistry included: caspase-3 (rabbit, 1:500; Cell Signaling), Ki67 (rat, 1:500; Invitrogen), Krox20 (rabbit, 1:500; kindly provided by Dr. Dies Meijer, University of Edinburgh), MAG (mouse, 1:500; Abcam), MBP (mouse, 1:1000; Covance), P0 (chicken, 1:3000; Aves), Sox2 (rabbit, 1:500; Millipore), and Sox9 (rabbit, 1:500; Cell Signaling Technology). Quantification of myelin protein immunoreactivity and marker-positive cells was performed using MetaMorph software (Molecular Devices). Integrated fluorescence intensity was calculated as the product of the mean pixel intensity and the stained area. To ensure consistent detection thresholds and avoid inclusion of low-intensity background pixels, the same fluorescence threshold was applied to all sections from each nerve. Background fluorescence was subtracted for all imaging channels prior to quantification. Analyses were conducted on pooled data from a minimum of four sciatic nerves per experimental group. For each nerve, at least 12 tissue sections were analyzed using a stereological sampling strategy. DAPI-positive nuclei were used to determine the total number of cells per field, which served as the denominator for calculating the percentage of marker-positive cells.

### Rotarod test

Coordinated motor performance was assessed using a rotarod apparatus following the standardized EMPReSS protocol (European Mouse Phenotyping Resource of Standardized Screens). Mice were acclimated to the testing room for at least 30 min prior to testing to minimize stress-related variability. Each animal was placed on a rotating rod under two testing protocols: (1) an accelerating protocol in which the rotation speed increased from 5 rpm to 40 rpm over a 5-min interval, and (2) a constant-speed protocol in which the rod rotated at 20 rpm for a maximum of 10 min. The latency to fall (time until the mouse dropped from the rod) was recorded for each trial using an automated timer integrated into the apparatus. To ensure reliability, each mouse completed three consecutive trials per protocol, spaced 20 min apart to allow recovery and reduce fatigue. Between trials, mice were returned to their home cages. A minimum of eight mice per experimental group were evaluated, and all testing was performed during the light phase under controlled environmental conditions (temperature, humidity, and noise). The apparatus was cleaned with 70% ethanol between trials to eliminate olfactory cues, and experimenters were blinded to group allocation to minimize bias.

### Western blot

Sciatic nerves were homogenized in lysis buffer containing 50 mM Tris–HCl (pH 8.0), 150 mM NaCl, 1% (w/v) Triton X-100, 0.5% (w/v) sodium deoxycholate, 0.1% (w/v) SDS, 1 mM PMSF, 1 mM NaF, 1 mM sodium orthovanadate, 1 mM AEBSF, and protease inhibitors including aprotinin (10 μg/mL), leupeptin (10 μg/mL), and pepstatin (10 μg/mL). Total protein concentrations were quantified using a bicinchoninic acid (BCA) assay. Equal amounts (20 μg) of protein were loaded onto NuPAGE Novex 4–12% Bis-Tris gels (Invitrogen) and transferred to polyvinylidene difluoride (PVDF) membranes. Membranes were blocked for 2 h at room temperature in PBS containing 5% non-fat milk and 0.2% Tween-20, followed by overnight incubation at 4 °C with primary antibodies. Detection was performed using horseradish peroxidase-conjugated secondary antibodies (GE Healthcare) and enhanced chemiluminescence (ECL; GE Healthcare). Fluorescent bands were imaged using a C-Digit Blot Scanner (LI-COR) and quantified using Image Studio Software (LI-COR). Primary antibodies: CNP (mouse, 1:3000; NeoMarkers), MBP (mouse, 1:1000; Covance), and α -tubulin (mouse; 1:10,000; Proteintech).

### Transmission electron microscopy

Sciatic nerves were dissected from mice and fixed in 2% glutaraldehyde overnight. Samples were then processed for resin embedding, sectioned into ultrathin slices, and stained with uranyl acetate and lead citrate. Imaging was performed using a Tecnai F20 transmission electron microscope (FEI). Quantitative ultrastructural analysis included measurements of the g-ratio and the percentage of myelinated axons. For each nerve, a minimum of 200 individual fibers and approximately 1,000 axons were evaluated. Image analysis was conducted using MetaMorph software (Molecular Devices) in a semi-automated manner. In addition, 100 randomly selected Remak bundles per nerve were classified based on established criteria (Feltri et al., 2016). At least six nerves per experimental group were included in the analysis.

### Statistical analysis

For single between-group comparisons, nested unpaired Student’s *t*-tests were performed using a 95% confidence interval. Multiple group comparisons were analyzed using nested one-way ANOVA followed by Bonferroni’s *post hoc* test to identify pairwise differences. All statistical analyses were conducted using GraphPad Prism software. Statistical significance was defined as *p* < 0.05 (two-tailed). Data are presented as mean ± SEM. Morphological and biochemical endpoints were assessed using a minimum of four animals per condition, with at least 12 slices per animal. Behavioral assays included a minimum of eight animals per group.

## Results

### Expression and activation of hM3Dq receptors in Schwann cells

**Figure 1:**
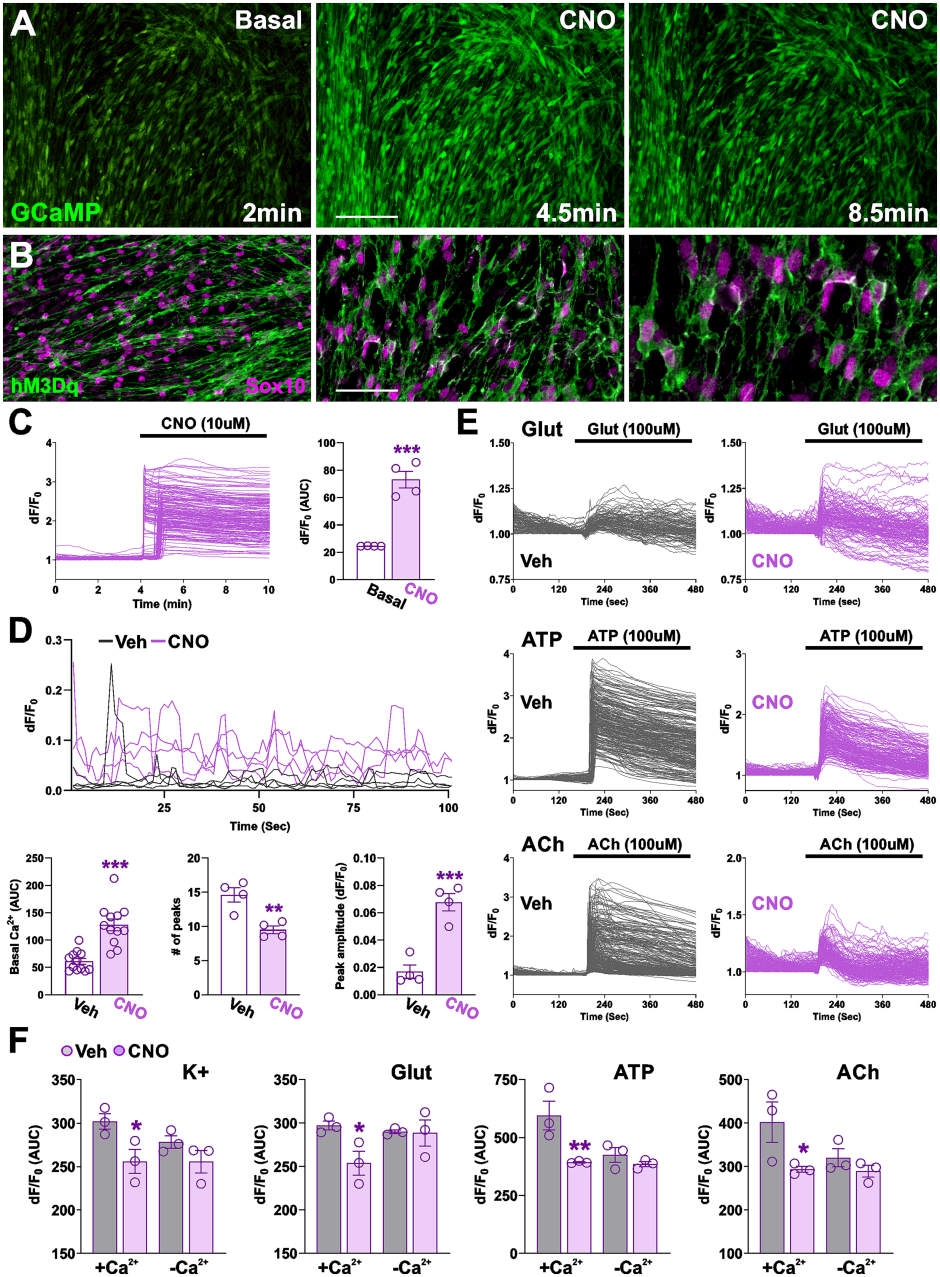
Expression and functional validation of hM3Dq in SCs within DRG co-culture. **(A)** CNO stimulation of hM3Dq-expressing SCs in DRG co-culture visualized using GCaMP fluorescence. Representative images of basal and CNO-stimulated cells are shown. Scale bar = 80 μm. **(B)** Immunocytochemical confirmation of hM3Dq expression in SCs using HA-tag and Sox10 antibodies. Representative images are shown. Scale bars = 60 μm, 40 μm, and 20 μm from left to right. **(C)** Functional validation of hM3Dq activation in SCs, demonstrated by an increase in GCaMP dF/F₀ following initial CNO stimulation. **(D)** Quantification of basal Ca^2+^ levels, peak frequency, and peak amplitude in SCs after 3 days of CNO treatment. Representative Ca^2+^ traces from individual vehicle- and CNO-treated SCs are shown. Data were collected from at least four independent biological replicates per condition, with approximately 100 cells analyzed per replicate. **(E,F)** SCs pre-treated with CNO for 3 days were subsequently stimulated with potassium (50 mM), glutamate (100 μM), ATP (100 μM), or acetylcholine (100 μM) in either Ca^2+^-free or Ca^2+^-containing media. **(E)** Representative Ca^2+^ traces of vehicle- and CNO-treated SCs stimulated with glutamate, ATP, and acetylcholine in the presence of extracellular Ca^2+^ are shown. Each trace represents a single cell; dF/F₀ measurements were recorded over a 480-s period from approximately 300 individual cells. **(F)** Bar graphs display changes in GCaMP fluorescence (dF/F₀) during stimulation, quantified as area under the curve (AUC). Dots represent values from independent biological replicates, each analyzing approximately 100 cells. Data are presented as mean ± SEM. Statistical significance: *p < 0.05; **p < 0.01; ***p < 0.001 versus corresponding vehicle control.

To target hM3Dq receptor expression specifically in Schwann cells (SCs), transgenic mice were generated by crossing hemizygous hM3Dq mice with hemizygous Sox10-i*Cre*ER^T2^ mice. The Sox10-i*Cre*ER^T2^ line expresses a tamoxifen-inducible *Cre* recombinase under the control of the Sox10 promoter, restricting *Cre* activity to SCs within the peripheral nervous system (PNS) (McKenzie et al., 2014). For clarity, conditional hM3Dq mice (hM3Dq^+/−^, Sox10-i*Cre*ER^T2 +/−^) will hereafter be referred to as *Sox10-hM3Dq*. Initial *in vitro* experiments were conducted using dorsal root ganglion (DRG) co-cultures prepared from Sox10-hM3Dq embryos. To determine how hM3Dq activation influences SC Ca^2+^ dynamics, DRG co-cultures were transduced with an adenoviral vector encoding the Ca^2+^ indicator GCaMP6m. Changes in intracellular Ca^2+^ within SC somas were monitored by measuring GCaMP6m fluorescence intensity (dF/F₀). Acute CNO application triggered robust Ca^2+^ transients in approximately 98% of SCs, with response kinetics consistent with classical intracellular Ca^2+^ release patterns (Figures 1A,C). Immunostaining further revealed that ~97% of Sox10-expressing cells in DRG co-cultures were positive for hM3Dq and displayed the characteristic morphology of immature SCs (Figure 1B). These findings confirm high recombination efficiency and functional expression of the hM3Dq receptor in SCs.

To evaluate the effects of prolonged hM3Dq activation on SC Ca^2+^ dynamics, DRG cultures were treated with CNO for three consecutive days. Compared to vehicle-treated controls, CNO-treated SCs exhibited a significant elevation in resting Ca^2+^ levels, indicating persistent hM3Dq activation (Figure 1D). Spontaneous Ca^2+^ oscillatory activity was monitored over a 10-min recording period in the same cells. In CNO-treated SCs, the frequency of spontaneous Ca^2+^ transients was reduced, whereas their amplitude was markedly increased, suggesting enhanced mobilization of intracellular Ca^2+^ stores or altered channel dynamics (Figure 1D). To further investigate channel and receptor function, SCs were stimulated with high K^+^ (to induce membrane depolarization), glutamate, ATP, or acetylcholine. In the presence of extracellular Ca^2+^, these assays measured both Ca^2+^ influx through plasma membrane channels and Ca^2+^ release from internal stores. Under these conditions, CNO-treated SCs showed a significantly smaller Ca^2+^ increase compared to controls (Figures 1E,F). In contrast, when experiments were performed in Ca^2+^-free media—where only Ca^2+^ release from internal stores is assessed—no differences were observed between groups (Figure 1F). These results indicate that hM3Dq activation primarily impairs ionotropic Ca^2+^ channel activity rather than metabotropic receptor signaling. Collectively, these findings demonstrate that hM3Dq activation elevates basal Ca^2+^ levels and increases the amplitude of spontaneous Ca^2+^ oscillations, while concurrently suppressing Ca^2+^ influx mediated by voltage- and ligand-gated channels in SCs.

**Figure 2:**
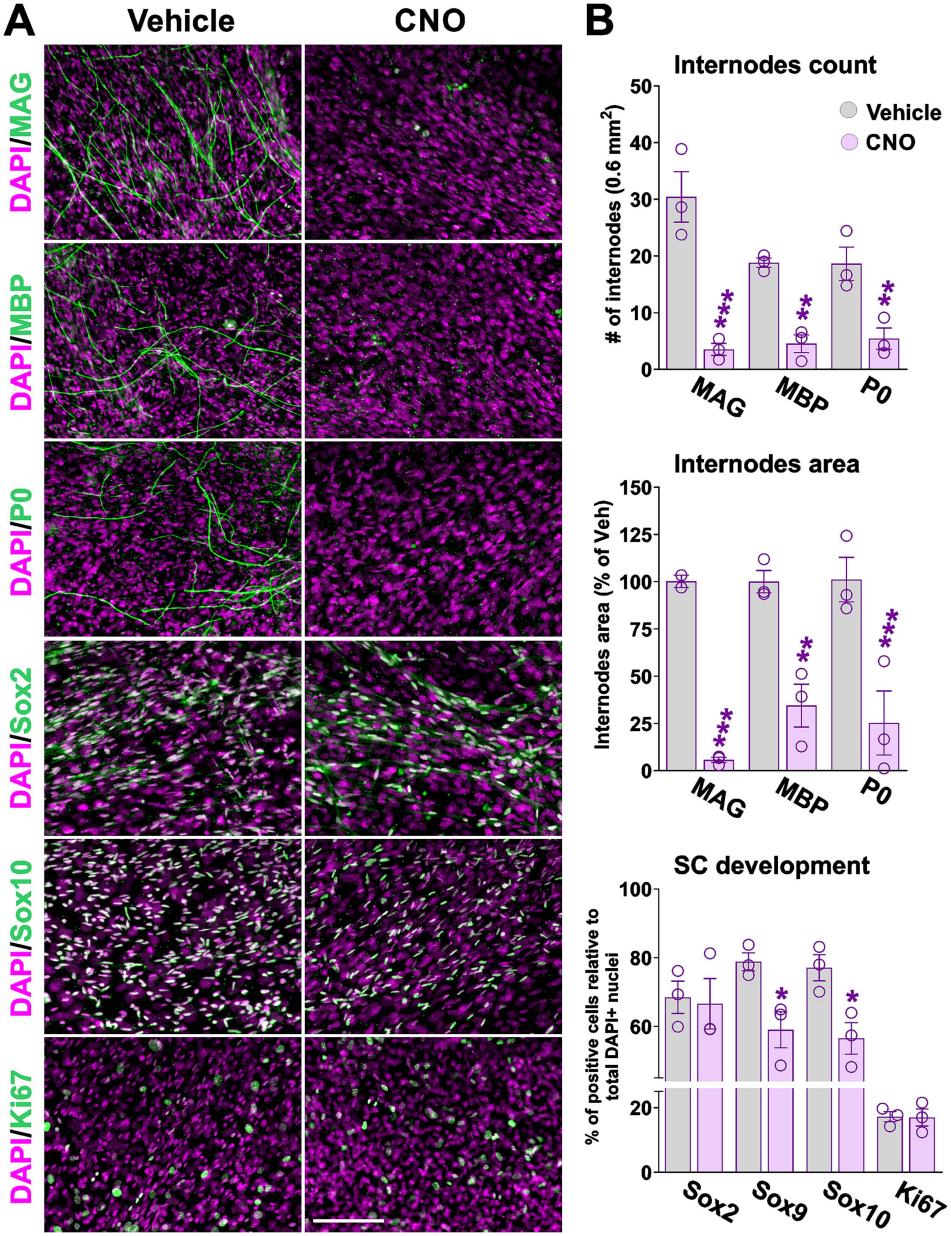
Activation of hM3Dq *in vitro* suppresses myelin synthesis and impairs SC maturation. To induce *Cre*-mediated recombination, DRG co-cultures were treated with 4-hydroxytamoxifen for two consecutive days starting on day 5 post-plating. Activation of the hM3Dq receptor was achieved by administering CNO for 14 days, beginning on day 7. To promote SC maturation and myelination, cultures were supplemented with ascorbic acid starting on day 7, in parallel with CNO treatment. **(A)** Immunocytochemical staining of DRG co-cultures for myelin-associated proteins (MAG, MBP, P0), transcription factors Sox2 and Sox10, and the proliferation marker Ki67. Representative images for each marker are shown. Scale bar = 80 μm. **(B)** Myelin internode number and area were quantified using immunostaining for MAG, MBP, and P0. Additionally, the percentage of Sox2-, Sox9-, Sox10-, and Sox9/Ki67-positive cells was measured in vehicle- and CNO-treated cultures. Measurements were performed in at least 10 randomly selected regions across three independent biological replicates. Bar graphs show quantification results, with dots representing values from independent biological replicates. Data are presented as mean ± SEM. Statistical significance: *p < 0.05, **p < 0.01, ***p < 0.001 versus corresponding vehicle control.

We next examined how sustained hM3Dq activation affects SC differentiation and myelination. To model this, DRG co-cultures were treated with ascorbic acid to induce myelination and simultaneously exposed to CNO for 2 weeks, ensuring continuous hM3Dq activation during the differentiation period. Myelination was assessed by quantifying the number and total area of internodes positive for MAG, MBP, and P0—key structural components of the myelin sheath. SC identity and maturation were evaluated using Sox2, Sox9, and Sox10 immunostaining. These transcription factors were chosen for their distinct roles in SC biology: Sox10 is essential for lineage specification and initiation of myelination; Sox9 is expressed throughout development and persists in mature SCs, serving as a marker of overall SC density; and Sox2 marks immature SCs and acts as a negative regulator of myelination when its expression is sustained (Monk et al., 2015). Two-week CNO treatment nearly abolished internode formation, markedly reducing both the number and coverage area of MAG-, MBP-, and P0-positive segments (Figures 2A,B). In parallel, the proportion of Sox9- and Sox10-expressing cells decreased significantly, while Sox2-positive SCs remained unchanged, indicating a failure to progress toward mature SC identity (Figures 2A,B). SC proliferation, assessed by Ki67 staining within Sox9-positive cells, was unaffected, and no evidence of cell death—such as pyknotic nuclei—was observed in either the Sox10- or Sox9-positive cell populations (Figures 2A,B). Nuclear density (DAPI) also remained consistent across conditions (Figure 2A). Collectively, these findings demonstrate that sustained hM3Dq activation strongly inhibits SC maturation and myelination during *in vitro* differentiation, without impacting cell proliferation or survival.

### hM3Dq activation inhibits SC maturation and myelination in young mice

**Figure 3:**
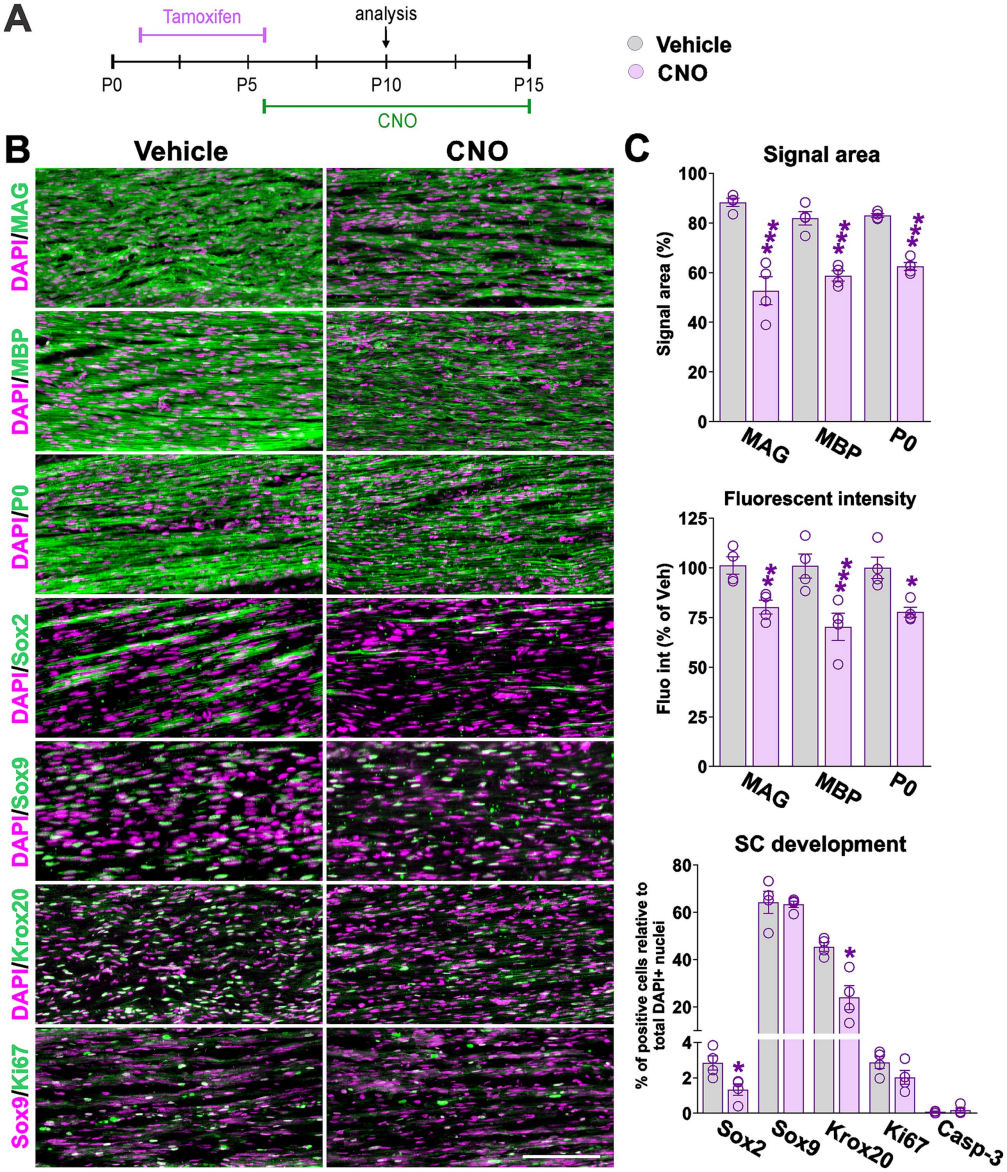
hM3Dq activation in young mice inhibits SC maturation and myelination. **(A)** Experimental timeline: mice were administered tamoxifen at postnatal day 2 (P2) to induce *Cre* recombinase activity, followed by daily treatment with CNO or vehicle from P6 to P15. Sciatic nerves were harvested at P10 for analysis. **(B)** Immunohistochemical staining of sciatic nerve sections for myelin-associated proteins (MAG, MBP, P0), transcription factors (Sox2, Sox9, Krox20), and the proliferation marker Ki67. Representative images for each marker are shown. Scale bar = 80 μm. **(C)** Quantification of signal area and fluorescence intensity for MAG, MBP, and P0, expressed as percentage relative to vehicle-treated controls. The percentage of Sox2-, Sox9-, Krox20-, Sox9/Ki67-, and caspase-3-positive cells was also calculated. Bar graphs show quantification results, with dots representing values from independent sciatic nerves. For all markers, a minimum of four sciatic nerves were analyzed, with at least 12 tissue sections examined per nerve. Data are presented as mean ± SEM. Statistical significance: *p < 0.05, **p < 0.01, ***p < 0.001 versus corresponding vehicle control.

To investigate SC development *in vivo*, Sox10-hM3Dq mice received tamoxifen injections from postnatal day (P) 2–6 and were treated daily with CNO for 10 days starting at P6 (Figure 3A). CNO is an inert compound that activates only DREADDs receptors. Importantly, we have previously reported that CNO administration does not affect CNS myelination in the absence of hM3Dq expression (Cheli et al., 2026). At P10, immunohistochemical analysis of sciatic nerves revealed a significant reduction in myelination in CNO-treated animals compared to vehicle controls, as assessed by myelin protein expression (MAG, MBP, P0) and stereological measurements (Figures 3B,C). Both the signal area—defined as the spatial extent of pixels exceeding a specified threshold—and fluorescence intensity—calculated as the product of mean pixel intensity and stained area (reflecting integrated signal)—were quantified. On average, the signal area of myelin proteins was reduced by approximately 40%, while fluorescence intensity decreased by 25% relative to vehicle-treated nerves (Figures 3B,C). Further analysis revealed a reduction in Sox2-positive cells in CNO-treated nerves, whereas the numbers of Sox9-positive cells and Sox9/Ki67 double-positive cells remained unchanged (Figures 3B,C). Consistent with impaired differentiation, the percentage of Krox20-positive SCs—a transcription factor essential for myelinating SC maturation (Monk et al., 2015)—was markedly reduced. To determine whether these changes were accompanied by cell loss, we assessed apoptotic cell death. No differences were detected in the percentage of active caspase-3-positive cells in CNO-treated nerves (Figure 3C). Total DAPI-positive cell counts per field/nerve section were likewise unchanged (data not shown), indicating that overall SC numbers were not affected by hM3Dq activation. Together—and consistent with our *in vitro* findings—these results demonstrate that hM3Dq activation disrupts SC progression toward a myelinating phenotype without altering proliferation or inducing cell death.

**Figure 4:**
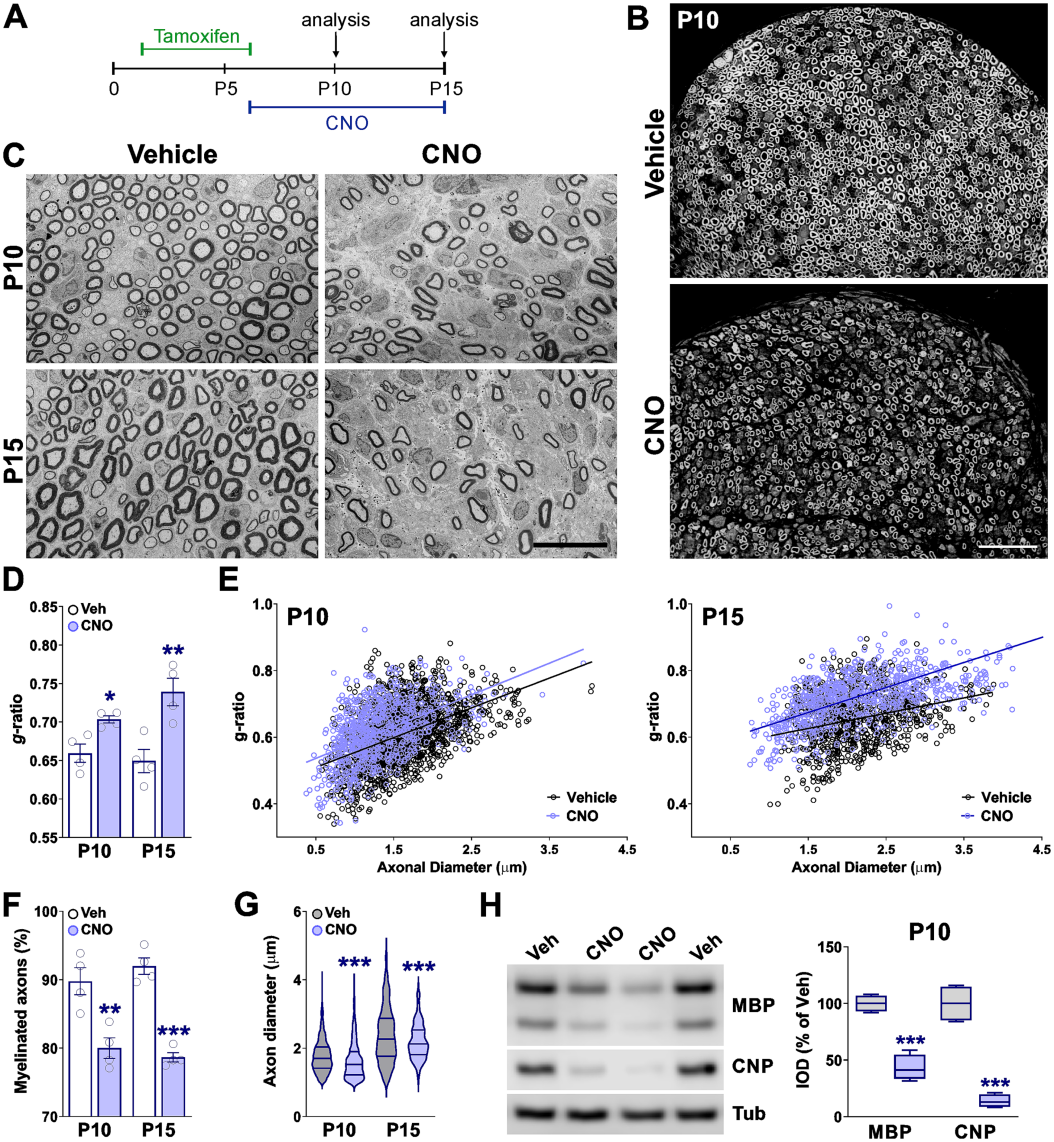
Ultrastructural analysis of sciatic nerves at P10 and P15 following hM3Dq activation. **(A)** Experimental timeline: mice were administered tamoxifen at postnatal day 2 (P2) to induce *Cre* recombinase activity, followed by daily treatment with CNO or saline (vehicle) from P6 to P15. Sciatic nerves were harvested at P10 and P15 for analysis. **(B)** Representative semi-thin sections of sciatic nerves from vehicle- and CNO-treated animals at P10. Scale bar = 20 μm. **(C)** Representative electron micrographs of sciatic nerves from vehicle- and CNO-treated animals at P10 and P15. Scale bar = 6 μm. **(D,E)** Quantification of myelin thickness using mean g-ratio values and scatter plots of g-ratio versus axon diameter in vehicle- and CNO-treated animals at P10 and P15. Regression lines with 95% confidence intervals are shown. **(F,G)** Percentage of myelinated axons and mean diameter of myelinated axons in vehicle- and CNO-treated animals at both time points. Four sciatic nerves per experimental condition were analyzed, with more than 200 fibers quantified per nerve. **(H)** Western blot analysis of total protein extracted from sciatic nerves at P10 to assess expression levels of MBP and CNP. *α*-Tubulin was used as a loading control. Data from four independent experiments are summarized based on relative band intensities and expressed as percentage of vehicle control. Data are presented as mean ± SEM. Statistical significance: *p < 0.05, **p < 0.01, ***p < 0.001 versus corresponding vehicle control.

To validate the immunohistochemical observations, we examined myelin ultrastructure using transmission electron microscopy, which revealed pronounced deficits in CNO-treated mice. At both P10 and P15 (Figure 4A), sciatic nerves exhibited significantly thinner myelin sheaths—reflected by an increased g-ratio—and a reduced number of myelinated axons compared to vehicle-treated controls (Figures 4B–G). Myelin thinning was observed across axons of all calibers, indicating a global impairment rather than a size-specific effect. The proportion of myelinated axons was approximately 10% lower in CNO-treated nerves at both time points (Figures 4E,F), and the mean diameter of myelinated axons was significantly smaller, suggesting delayed myelination (Figure 4G). At the molecular level, Western blot analysis of sciatic nerve extracts at P10 confirmed reduced expression of key myelin proteins, including MBP and 2′,3′-cyclic-nucleotide 3′-phosphodiesterase (CNP), in CNO-treated mice (Figure 4H). Together, these findings demonstrate that sustained hM3Dq activation in SCs profoundly impairs myelination *in vivo*, resulting in thinner myelin sheaths, fewer myelinated axons, and decreased expression of essential myelin components.

**Figure 5:**
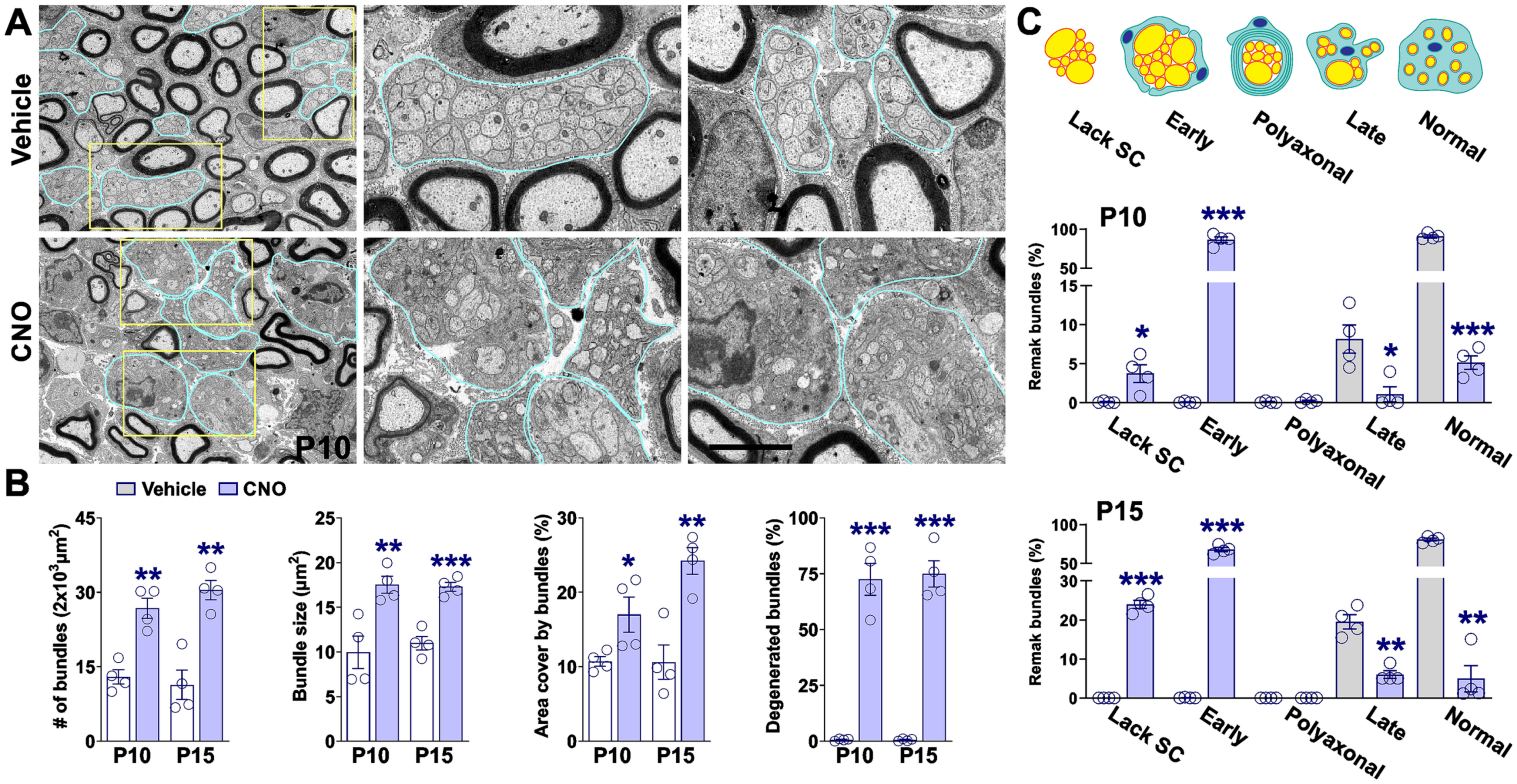
Axonal sorting in the sciatic nerve following hM3Dq activation. **(A)** Representative electron micrographs of sciatic nerves from vehicle- and CNO-treated mice at P10. Remak bundles are outlined in light blue. Yellow boxes indicate zoom-in regions. Scale bar = 6 μm; 2 μm for zoom-in regions. **(B)** Quantification of the total number of Remak bundles, bundle size, the area they occupy, and the percentage of degenerated bundles in vehicle- and CNO-treated nerves at P10 and P15. **(C)** Classification of Remak bundles into five categories based on criteria from Feltri et al. (2016). The distribution of Remak bundle types was assessed in vehicle- and CNO-treated mice at both time points. Four sciatic nerves per experimental condition were analyzed, with more than 100 Remak bundles quantified per nerve. Data are presented as mean ± SEM. Statistical significance: *p < 0.05, **p < 0.01, ***p < 0.001 versus corresponding vehicle control.

Remak bundles are specialized structures in peripheral nerves where non-myelinating SCs ensheath groups of small-caliber axons, providing trophic support and maintaining axonal integrity. These bundles are essential for proper nerve function, and their abundance and morphology can serve as indicators of developmental progression or pathology. An increase in unmyelinated axon bundles often reflects delayed or impaired myelination (Feltri et al., 2016). In our study, both the total number and the area occupied by unmyelinated axon bundles were significantly higher in CNO-treated mice at P10 and P15 compared to vehicle controls (Figures 5A,B). At both time points, bundle numbers increased by approximately 50%, and the area occupied by these axon groups rose from ~18% at P10 to ~24% at P15, whereas control nerves consistently showed ~10% (Figures 5A,B). Moreover, degenerated Remak bundles—characterized by disorganized axons, axonal swelling or fragmentation, and vacuolization—were markedly more prevalent in CNO-treated nerves. Quantitative analysis revealed that over 75% of bundles in CNO-treated mice exhibited abnormal morphology at both time points (Figures 5A,B).

To further characterize bundle organization, we applied the classification system described by Feltri et al. (2016) (Figure 5C). CNO-treated mice at P10 and P15 exhibited a marked increase in the proportion of early-stage bundles, accompanied by a pronounced reduction in late-stage and morphologically normal bundles (Figure 5C). Although polyaxonal bundles were not observed, we detected a substantial rise in bundles lacking associated SCs in CNO-treated nerves (Figure 5C). Together, these findings indicate that sustained hM3Dq activation delays SC maturation, leading to impaired myelination and abnormal Remak bundle morphology.

### hM3Dq activation induces myelin loss and neurodegeneration in the adult PNS

**Figure 6:**
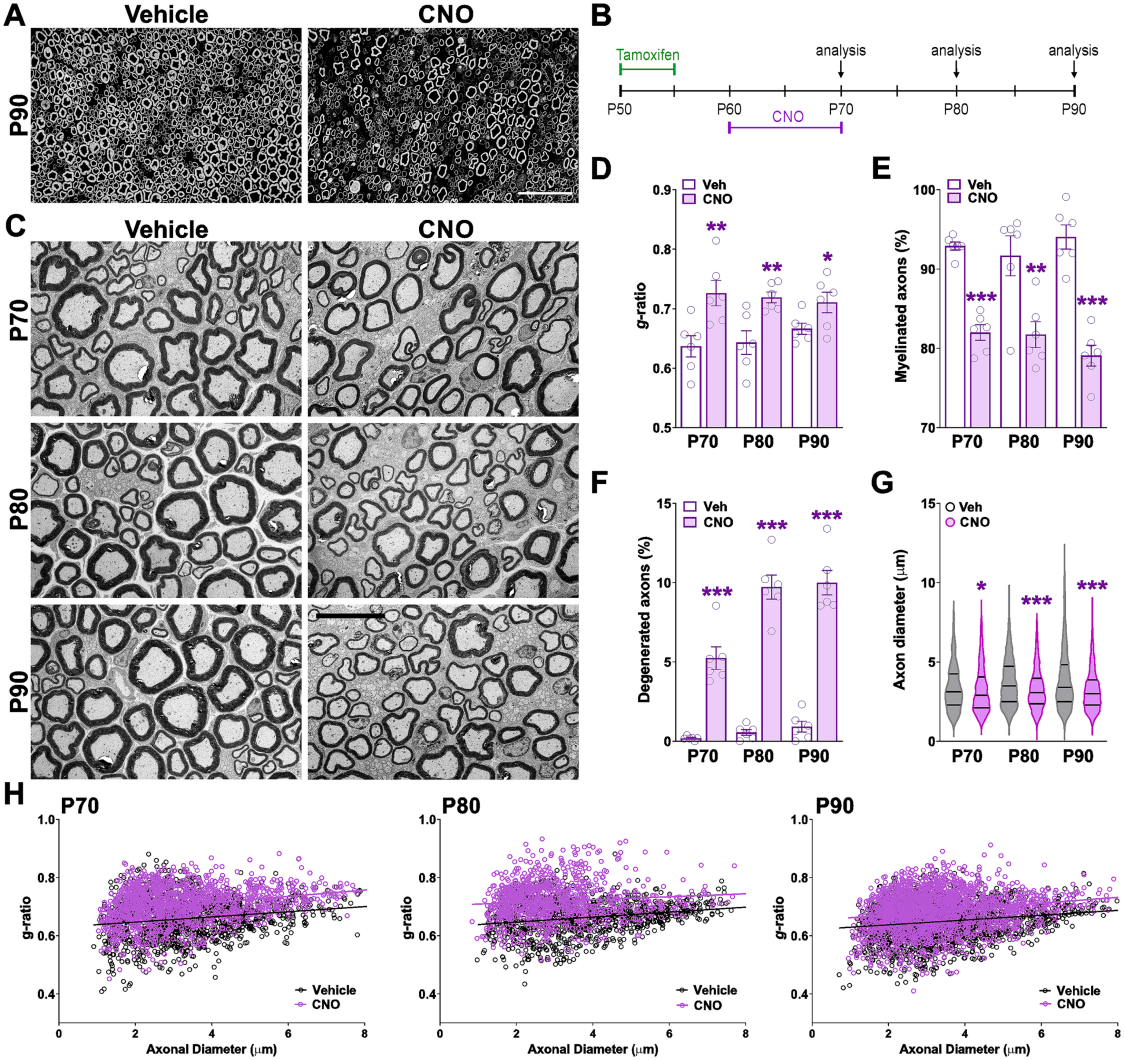
Ultrastructural analysis of adult sciatic nerves after hM3Dq activation. **(A)** Representative semi-thin sections of sciatic nerves from vehicle- and CNO-treated animals at P90. Scale bar = 20 μm. **(B)** Experimental timeline: mice were administered tamoxifen at postnatal day 50 (P50) to induce *Cre* recombinase activity, followed by daily treatment with CNO or saline (vehicle) from P60 to P70. Sciatic nerves were harvested at P70, P80, and P90 for analysis. **(C)** Representative electron micrographs of sciatic nerves from vehicle- and CNO-treated animals at P70, P80, and P90. Scale bar = 6 μm. **(D–G)** Quantification of myelin thickness using mean g-ratio values, percentage of myelinated axons, percentage of degenerated axons, and mean diameter of myelinated axons in vehicle- and CNO-treated animals at all time points. **(H)** Scatter plots of g-ratio versus axon diameter in vehicle- and CNO-treated animals at P70, P80, and P90. Regression lines with 95% confidence intervals are shown. Six sciatic nerves per experimental condition were analyzed, with more than 200 fibers quantified per nerve. Data are presented as mean ± SEM. Statistical significance: *p < 0.05, **p < 0.01, ***p < 0.001 versus corresponding vehicle control.

**Figure 7:**
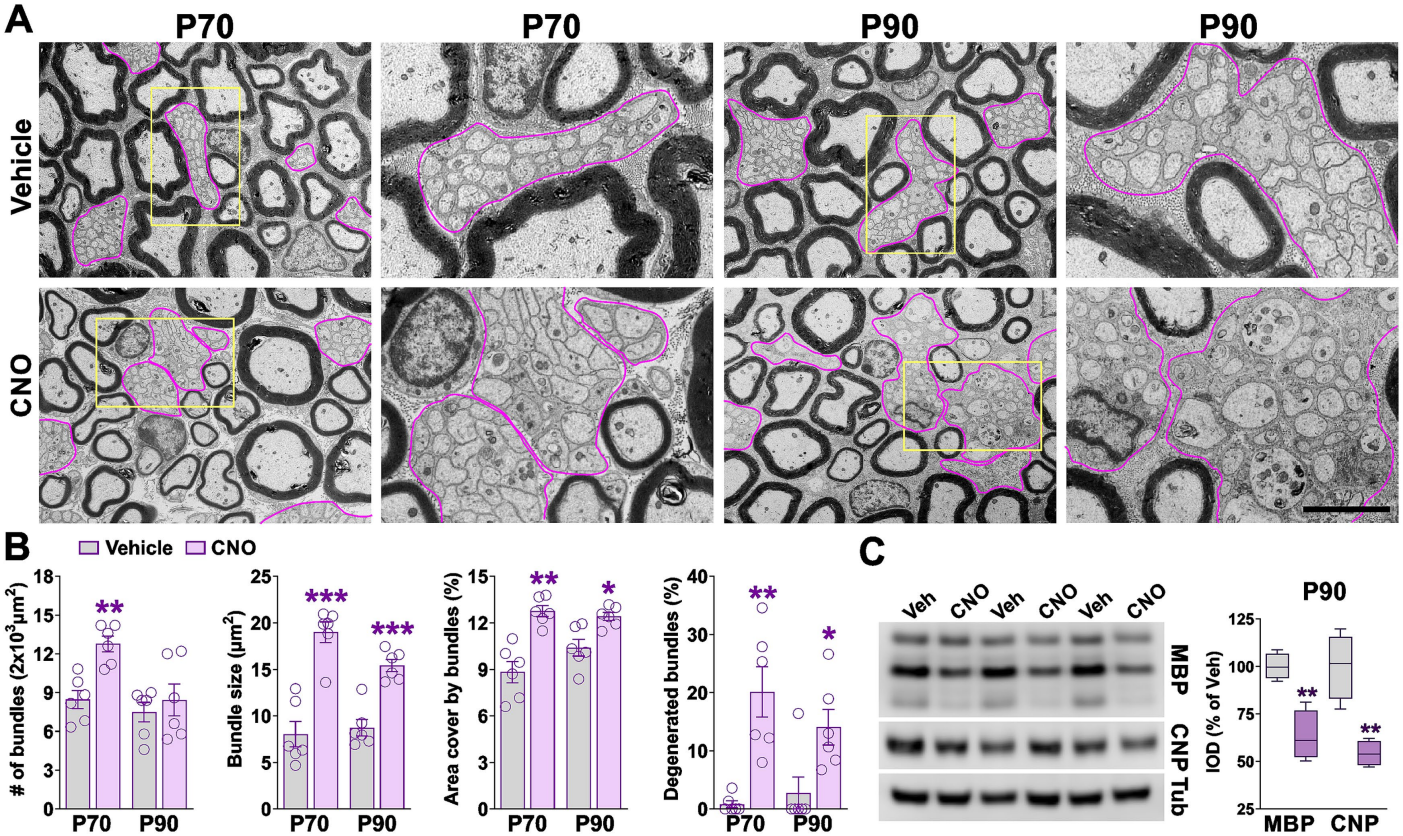
Remak bundles in the adult hM3Dq sciatic nerve. **(A)** Representative electron micrographs of sciatic nerves from vehicle- and CNO-treated mice at P70 and P90. Remak bundles are outlined in magenta. Yellow boxes indicate zoom-in regions. Scale bar = 6 μm; 2 μm for zoom-in regions. **(B)** Quantification of the total number of Remak bundles, bundle size, the area they occupy, and the percentage of degenerated bundles in vehicle- and CNO-treated nerves at P70 and P90. Six sciatic nerves per experimental condition were analyzed, with more than 100 Remak bundles quantified per nerve. **(C)** Western blot analysis of total protein extracted from sciatic nerves at P90 to assess expression levels of MBP and CNP. α-tubulin was used as a loading control. Data from four independent experiments are summarized based on relative band intensities and expressed as percentage of vehicle control. Data are presented as mean ± SEM. Statistical significance: *p < 0.05, **p < 0.01 versus corresponding vehicle control.

To investigate the effects of hM3Dq activation in the adult PNS, Sox10-hM3Dq mice were administered tamoxifen at postnatal day 50 (P50) and subsequently treated with CNO daily from P60 to P70 (Figure 6B). Electron microscopy analysis of sciatic nerves at P70, P80, and P90 revealed increased g-ratios and a reduced percentage of myelinated axons in CNO-treated animals (Figures 6A,C–E,H). G-ratio values plotted against axonal diameter indicated that axons of all calibers were affected (Figure 6H). While reductions in myelin thickness were consistent across time points, the decline in the proportion of myelinated axons progressively worsened from P70 to P90, falling below 80% at the latest time point (Figures 6D,E). In parallel, the diameter of myelinated axons was significantly reduced in CNO-treated sciatic nerves (Figure 6G). Signs of axonal degeneration—including axonal swelling, mitochondrial abnormalities, and the presence of autophagic vacuoles—were prominent in CNO-treated mice (Figure 6F). The percentage of abnormal axons increased significantly over time, exceeding 10% by P90 (Figure 6F). Furthermore, CNO-treated mice at P70 and P90 exhibited a significant increase in the number, size, and total area of unmyelinated axon bundles (Figures 7A,B). Notably, over 20% of these bundles displayed abnormal morphology, including axonal swelling, fragmentation, and vacuolization at both time points (Figures 7A,B). Western blot analysis of sciatic nerve lysates at P90 confirmed these findings, showing reduced expression of key myelin proteins including MBP and CNP (Figure 7C). These findings demonstrate that hM3Dq activation in adult SCs induces progressive myelin degradation and neurodegeneration in peripheral nerves.

**Figure 8:**
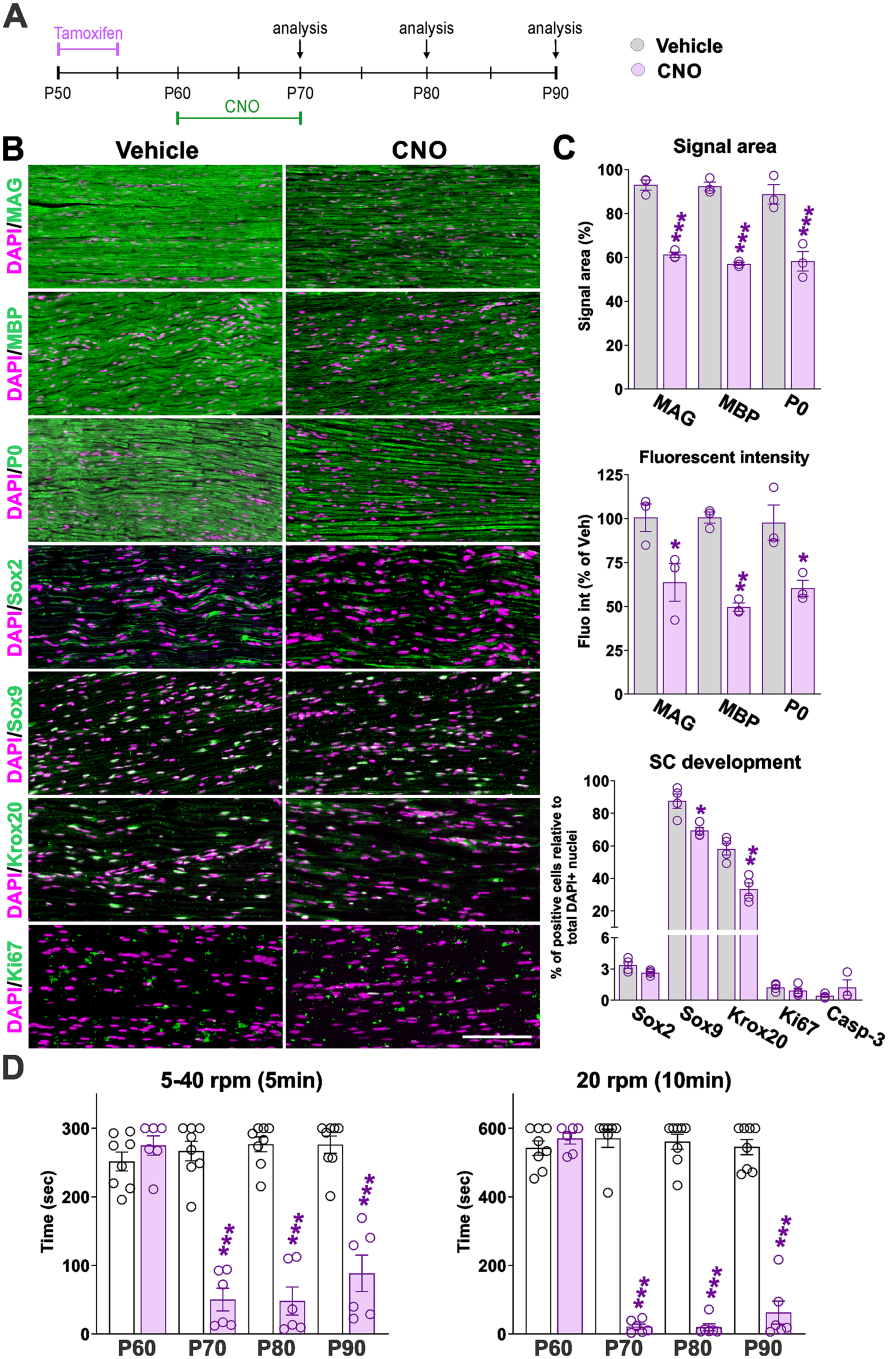
hM3Dq activation in adult sciatic nerves induces demyelination. **(A)** Experimental timeline: mice were administered tamoxifen at postnatal day 50 (P50) to induce *Cre* recombinase activity, followed by daily treatment with CNO or saline (vehicle) from P60 to P70. Sciatic nerves were harvested at P90 for analysis. **(B)** Immunohistochemical staining of sciatic nerve sections for myelin-associated proteins (MAG, MBP, P0), transcription factors (Sox2, Sox9, Krox20), and the proliferation marker Ki67. Representative images for each marker are shown. Scale bar = 80 μm. **(C)** Quantification of signal area and fluorescence intensity for MAG, MBP, and P0, expressed as percentage relative to vehicle-treated controls. The percentage of Sox2-, Sox9-, Krox20-, Sox9/Ki67-, and caspase-3-positive cells was also calculated. Bar graphs show quantification results, with dots representing values from at least three independent sciatic nerves. For all markers, at least 12 tissue sections were analyzed per nerve. **(D)** Motor coordination in adult mice assessed using the rotarod test. Latency to fall was measured at postnatal days P60, P70, P80, and P90 using two protocols: (1) An accelerating protocol with rotation speed increasing from 5 to 40 rpm over 5 min, and (2) a constant-speed protocol with rotation at 20 rpm for a maximum of 10 min. At least eight mice were tested per experimental group. Data are presented as mean ± SEM. Statistical significance: *p < 0.05, **p < 0.01, ***p < 0.001 versus corresponding vehicle control.

Consistent with previous results, immunohistochemical analysis revealed a marked reduction in myelin content within the sciatic nerve following CNO treatment (Figures 8A–C). Quantification of myelin-associated proteins—MAG, MBP, and P0—at P90 confirmed significantly decreased myelination in CNO-treated mice compared to vehicle controls (Figures 8B,C). On average, the signal area of myelin proteins was reduced by ~40%, while fluorescence intensity declined by more than 45% relative to controls (Figures 8B,C). Although the proportions of Sox2-positive cells and Sox9/Ki67 double-positive cells were unchanged, we observed a notable reduction in Sox9-positive cells (Figures 8B,C). In addition, the number of Krox20-expressing SCs was markedly decreased, indicating a diminished population of myelinating SCs in mature peripheral nerves (Figures 8B,C). Despite these shifts in SC marker expression, the proportion of active caspase-3–positive cells remained unchanged in CNO-treated nerves (Figure 8C), and the density of DAPI-positive nuclei also remained constant (data not shown). Together, these findings indicate that hM3Dq activation does not increase SC death in adult sciatic nerves.

Because demyelination disrupts ion channel distribution and impairs action potential conduction, leading to motor deficits (Coggan et al., 2010; Devaux and Scherer, 2005), we assessed motor coordination using the rotarod test under constant and accelerating speed protocols (Figure 8D). Vehicle-treated mice maintained stable performance across all time points, whereas CNO-treated mice exhibited a significant reduction in latency to fall at P70, P80, and P90, indicating impaired motor coordination following hM3Dq activation in mature SCs (Figure 8D). Collectively, these results demonstrate that hM3Dq activation in adult peripheral nerves disrupts SC homeostasis, compromises myelin maintenance, and leads to functional motor deficits.

## Discussion

We used *Cre*-dependent expression of the excitatory DREADD hM3Dq to achieve temporal control of the canonical Gq–PLC–IP3–Ca^2+^ signaling cascade in SCs. Activation of hM3Dq triggered Ca^2+^ release from internal stores, increased the amplitude of spontaneous Ca^2+^ oscillations, and suppressed Ca^2+^ signaling through voltage-gated and ligand-gated channels for glutamate, ATP, and acetylcholine. During early postnatal development, hM3Dq activation in sciatic nerve SCs markedly delayed myelination and reduced the pool of mature SCs, whereas activation in adulthood caused myelin loss, peripheral nerve degeneration, and motor deficits. Across both *in vitro* and *in vivo* experiments, hM3Dq activation consistently decreased markers of mature SC identity without affecting proliferation or survival. *In vitro*, CNO treatment reduced the number of Sox9- and Sox10-positive SCs. *In vivo*, Krox20-positive SCs were significantly reduced in young sciatic nerves, and both Sox9- and Krox20-positive SCs were diminished in adult nerves. Together, these findings indicate that hM3Dq activation alters SC identity rather than causing SC loss, potentially through downregulation of transcription factors essential for SC maturation and myelin maintenance. Overall, these results demonstrate that SC Ca^2+^ signaling can be precisely manipulated using excitatory designer receptors, establishing hM3Dq as a powerful tool to modulate SC development and PNS myelination across life stages.

Notably, our results parallel observations in oligodendrocytes (Cheli et al., 2026), revealing conserved yet context-specific effects of hM3Dq-mediated signaling on myelinating glia. In both systems, hM3Dq activation induces intracellular Ca^2+^ release and disrupts maturation, leading to impaired myelination during development and demyelination in adulthood. However, the direction of Ca^2+^ channel modulation diverges: oligodendrocytes exhibit enhanced Ca^2+^ influx through voltage-gated and glutamate channels, whereas SCs show suppressed influx despite elevated basal Ca^2+^ and amplified spontaneous oscillations. Functionally, hM3Dq activation promotes OPC proliferation and delays differentiation in the CNS, while in the PNS it blocks SC maturation without affecting proliferation or survival. These findings underscore a shared vulnerability of myelinating glia to Ca^2+^ dysregulation, while highlighting lineage-specific mechanisms that shape their response to excitatory Gq signaling.

Neuronal activity is a critical regulator of SC development, with neurotransmitter release from peripheral axons influencing their proliferation, differentiation, and myelination (Stevens et al., 2004; Samara et al., 2013). These neuron–glia interactions persist throughout adulthood and are essential for maintaining axonal function, myelin integrity, and regeneration (Lopez-Verrilli and Court, 2012; Samara et al., 2013). Action potentials in peripheral neurons trigger vesicular release of ATP, glutamate, and acetylcholine (Samara et al., 2013; Vizi et al., 2010). SCs express both voltage-gated and ligand-gated Ca^2+^ channels, enabling them to sense and respond to neurotransmitter signals through intracellular Ca^2+^ dynamics (Stevens and Fields, 2000; Heredia et al., 2020). Transcriptomic and electrophysiological studies have identified multiple Ca^2+^-permeable channels and receptors in SCs, including purinergic (P2X, P2Y), glutamatergic (NMDA, AMPA, and mGluR), muscarinic (M2, M3), and voltage-gated channels (Cav1.2, Cav2.1) (Samara et al., 2013; Heredia et al., 2020; Colomar and Amédée, 2001). Purinergic signaling regulates SC proliferation and myelination, while activation of P2 receptors suppresses myelin protein expression and promotes a shift toward Remak bundle–like phenotypes (Faroni et al., 2014a, 2014b; Patritti-Cram et al., 2021). Conversely, glutamatergic signaling via mGluR2/3 supports SC proliferation and differentiation (Saitoh et al., 2016). Sustained neuronal activity also elevates extracellular K^+^, transiently depolarizing SC membranes and inducing Ca^2+^ influx through voltage-gated channels (Heredia et al., 2020; Larsen et al., 2016). Our findings show that prolonged hM3Dq activation reduces the activity of multiple Ca^2+^ channels, including voltage-gated, purinergic, and glutamatergic types. This widespread suppression suggests that sustained hM3Dq signaling profoundly alters SC physiology and diminishes their responsiveness to neurotransmitter cues, thereby impairing activity-dependent mechanisms essential for myelin formation and long-term maintenance. By limiting SCs’ ability to integrate neuronal signals, hM3Dq-mediated disruption not only delays developmental myelination but also destabilizes mature myelin, ultimately leading to structural degeneration and functional deficits.

Muscarinic acetylcholine receptors (mAChRs) play critical roles in regulating oligodendrocytes and SCs, influencing proliferation, differentiation, and responses to injury (De Angelis et al., 2012; Piovesana et al., 2022). In OPCs, M2 and M3 receptor subtypes predominate: M3 signaling promotes mitogenic activity, maintaining the progenitor pool, whereas M2 activation impairs OPC survival and maturation (Paez and Lyons, 2020). Notably, M3 receptor activity inhibits efficient remyelination in human and mouse OPCs, suggesting that its antagonism may enhance oligodendrocyte differentiation and myelin repair (Abiraman et al., 2015; Welliver et al., 2018). Similarly, in SCs, mAChRs modulate phenotype and differentiation (Loreti et al., 2006; Piovesana et al., 2020, 2022). Rat and human SCs express multiple mAChR subtypes, with M2 and M3 being most abundant; M2 activation suppresses proliferation and promotes differentiation toward a myelinating phenotype (Loreti et al., 2006; Piovesana et al., 2020, 2022, 2025). Mechanistically, M2 signaling—coupled to inhibitory Gi/o proteins—downregulates immature SC markers such as c-Jun and Notch-1 while upregulating promyelinating transcription factors like Sox10 and Krox20 to stabilize the myelinating phenotype (Piovesana et al., 2022). In contrast, M3 receptors couple to Gq proteins, activating PLC–IP3–Ca^2+^ signaling, which in other glial systems sustains progenitor states and delays maturation—effects likely shared by SCs. Our findings extend these observations by showing that Ca^2+^ signaling mediated by M3 receptors delays SC maturation and modulates axon–glia interactions during early development, while sustained activation in mature SCs induces demyelination and axonal degeneration. These results highlight acetylcholine as a key mediator of neuron–glia communication in the PNS and underscore mAChRs—particularly M3 receptors—as promising therapeutic targets for modulating glial behavior in demyelinating and neurodegenerative disorders.

The SCs are classified into myelinating (mSCs) and non-myelinating (nmSCs) subtypes. mSCs ensheath large-diameter axons in compact myelin to enable rapid saltatory conduction (Chen and Kukley, 2020), whereas nmSCs form Remak bundles that provide structural and trophic support to small-caliber axons (Oliveira et al., 2023). Radial sorting—a critical developmental process in the PNS—relies on axon–SC signaling, where SCs segregate large axons for myelination while smaller axons remain grouped in non-myelinating bundles (Berti et al., 2011; Faroni et al., 2014a; Feltri et al., 2016). The abundance and morphology of unmyelinated axon bundles often serve as indicators of pathological states, as increased bundle formation is associated with delayed or abnormal myelination (Feltri et al., 2016). Emerging evidence suggests that intracellular Ca^2+^ signaling intersects with pathways controlling SC phenotype and axon ensheathment, implying that Ca^2+^ dysregulation may contribute to abnormal Remak bundle morphology and stability (Numata et al., 2025). In our study, hM3Dq activation in SCs produced a significant increase in the number, size, and total area of unmyelinated axon bundles in the sciatic nerve during both early development and adulthood. Many of these bundles exhibited abnormal morphology, including axonal swelling, fragmentation, and vacuolization, indicating disrupted axon–glia communication. These structural abnormalities suggest that hM3Dq activation perturbs SC organization and compromises proper Remak bundle formation and maintenance throughout life. Furthermore, in this altered physiological state, some SCs may retract their processes or fail to maintain contact with small-caliber axons, giving rise to the increased number of Remak bundles lacking visible SC cytoplasm. Notably, these bundles retain extracellular matrix components and remnants of the SC basal lamina, consistent with structural persistence without active ensheathment rather than SC loss. Together, these findings indicate that Gq-mediated Ca^2+^ signaling plays a modulatory role in Remak bundle organization and stability, revealing a previously underappreciated mechanism governing axon–glia interactions in the PNS.

Ca^2+^ signaling in mature, myelinating SCs, mediated by muscarinic and purinergic receptors, is essential for maintaining myelin integrity and regulating its turnover in the adult PNS (Monk et al., 2009; Rochon et al., 2001; Castonguay and Robitaille, 2001). However, excessive intracellular Ca^2+^ can trigger cellular stress responses, leading to mitochondrial dysfunction, oxidative stress, and apoptosis, ultimately compromising myelination and SC viability. This Ca^2+^-induced toxicity is particularly relevant in peripheral neuropathies, where dysregulated Ca^2+^ signaling exacerbates damage (Yan et al., 2012). For example, PMP22 mutations associated with Charcot–Marie-Tooth (CMT) disease enhance store-operated Ca^2+^ channel activity via TrpC1 and STIM1, increasing intracellular Ca^2+^ and contributing to pathogenesis (Vanoye et al., 2019). Similarly, mutations in connexin 32—which mediates Ca^2+^ and metabolite exchange within non-compact myelin—are also linked to CMT (Scherer and Wrabetz, 2008). Our findings demonstrate that sustained hM3Dq activation in mature SCs leads to progressive myelin degradation and axonal degeneration. Although hM3Dq activation suppresses Ca^2+^ influx through voltage- and ligand-gated channels, it elevates basal Ca^2+^ levels and amplifies spontaneous Ca^2+^ oscillations. Persistent intracellular Ca^2+^ elevation may induce Ca^2+^-dependent toxicity, triggering stress responses that result in mitochondrial dysfunction, energy depletion, and impaired myelin maintenance. These results underscore the importance of tightly regulated Ca^2+^ signaling for preserving myelin integrity and preventing demyelinating pathologies and peripheral neuropathies.

In conclusion, our findings demonstrate that chemogenetic activation of SCs via hM3Dq profoundly alters Ca^2+^ signaling, disrupts maturation, and impairs myelination during development, while inducing demyelination and axonal degeneration in adulthood. These results underscore the critical role of Ca^2+^ homeostasis in SC biology and establish excitatory DREADDs as powerful tools for probing and manipulating PNS myelination. While hM3Dq activation may also engage other Gq-coupled pathways, such as PKC and MAPK signaling, which were not dissected here, our data strongly implicate Ca^2+^ dysregulation as a central driver of these effects. Motor deficits observed in adult animals could reflect contributions from CNS demyelination, as previously reported (Cheli et al., 2026), but the robust PNS pathology highlights the relevance of this mechanism to peripheral nerve biology. Future studies should leverage transcriptomic profiling to distinguish Ca^2+^-dependent from Ca^2+^-independent pathways and explore whether these changes are reversible or therapeutically targetable, paving the way for novel strategies to modulate myelination in health and disease.

## Data Availability

The original contributions presented in the study are included in the article/Supplementary material, further inquiries can be directed to the corresponding author.
