# Supplementary figures and images for: Chemogenetic Schwann cell activation impairs early myelination and triggers adult demyelination in the peripheral nervous system
Source: Front Cell Neurosci. 2026 Feb 16;20:1771951. doi: 10.3389/fncel.2026.1771951 (PMC12950554; doi:10.3389/fncel.2026.1771951)

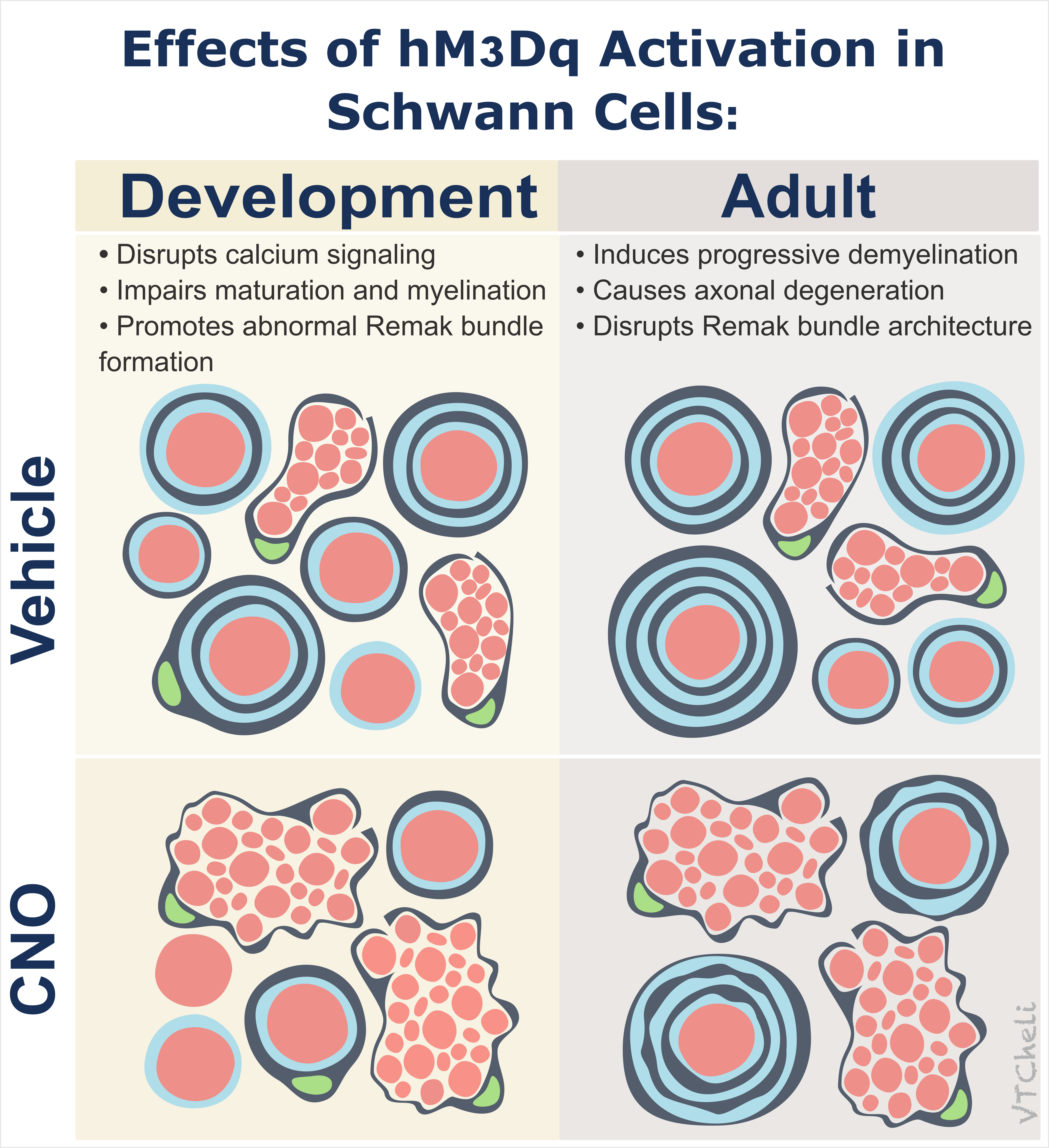

Supplement: Supplementary file 1 [file Image_1.PNG]
